# Case Report: Gas in the esophagus, stomach wall and portal vein with congenital hypertrophic pyloric stenosis

**DOI:** 10.3389/fped.2024.1348746

**Published:** 2024-02-08

**Authors:** Na Yao, Wenxin Zhang, Qi Gao, Chaoxiang Lu, Qi Wang

**Affiliations:** ^1^ Department of Nursing, The Affiliated Children Hospital of Xi'an Jiaotong University, Xi’an, Shaanxi Province, China; ^2^ Department of General Surgery, The Affiliated Children Hospital of Xi'an Jiaotong University, Xi’an, Shaanxi Province, China

**Keywords:** CHPS, pyloromyotomy, necrotizing enterocolitis, vomiting, vomiting without bile

## Abstract

**Background:**

CHPS dramatically affects infant growth and development and can even cause aspiration resulting from esophageal reflux. There is potential danger. CHPS is common, while CHPS with gas in the stomach wall and portal vein is rare. Gas in the stomach wall and portal vein are often the key features of more serious disease. It can be easily mistaken as a serious disease when patients with CHPS have gas in the stomach wall and portal vein.

**Case presentation:**

A 56-day-old baby was hospitalized for aspiration pneumonia after vomiting without bile for 20 days. Compared with vomiting, which is the most common symptom, pneumonia tends to attract more attention. Because of pneumonia, a chest CT scan was performed and revealed massive gas accumulation in the walls of the esophagus, stomach, and portal vein. Therefore, NEC was considered first and was treated conservatively for one week. However, the vomiting continued, and CHPS was confirmed by ultrasound. The delay in CHPS diagnosis was due to insufficient recognition of the signs of gas accumulation. Because of inexperience and lack of knowledge about CHPS with gastrointestinal pneumatosis, physicians failed to make an early accurate diagnosis. Case 2 was a 29-day-old male who was admitted to the hospital with vomiting without bile. He was examined by ultrasound, which revealed gas in the stomach wall and portal vein after admission to the hospital. No peritonitis was found after a detailed and comprehensive physical examination. Emergency life-threatening diseases such as NEC were quickly ruled out. He received surgery as soon as possible and had an uneventful recovery with no complications.

**Conclusion:**

CHPS may present with gas in the gastric or esophageal wall and portal vein, which is not a contraindication to surgery.

## Introduction

Congenital hypertrophic pyloric stenosis (CHPS) is a common digestive tract malformation in infants with an incidence of up to 0.4%. The fundamental cause is unknown, although a number of predisposing conditions, such as genetic factors, mechanical stimulation, and gastrointestinal hormone disorders, are recognized. Infants could suffer electrolyte disturbances, dehydration, malnutrition, weight loss, or even die if they do not receive an early diagnosis and effective treatment. Pneumatosis intestinalis and hepatic portal venous gas are normally observed in transmural infarction or necrotizing enterocolitis (NEC). Images of pneumatosis intestinalis or hepatic portal venous gas can be misdiagnosed as other life-threatening diseases. The signs are extremely rare in CHPS and are often easily misdiagnosed. It is obvious that these can be diagnosed with lots of doubts and fears. Some probably think diagnosis maybe is wrong, especially in in primary hospitals. It can be difficult to tell whether vomiting is from CHPS or NEC and enterobrosis, particularly in babies because they sometimes do not cooperate very well during a physical checkup. When the disease is severe, the baby's poor cooperation and inexperienced inspectors can affect obtaining the correct diagnosis; therefore, an early, quick and accurate diagnosis is usually not obtained. When patients have clinical symptoms of acute gastric mucosal hemorrhage, it can provide an effective basis for the diagnosis of NEC. It is indisputable that conservative treatment will inevitably take longer and actually prolong the disease. Therefore, the probably scheduled CHPS operation would be unexpectedly delayed. The situation could also get worse with delay of surgery.

Junior doctors or primary hospitals should pay more attention to the abdominal physical signs if such a case is encountered. Both cases were reported in our center and presented with symptoms of vomiting accompanied by gas in the esophagus, stomach wall and portal vein. We have to think about whether the CHPS were truly doubtless, and how to choose the most appropriate surgical technique, including deciding if laparoscopic surgery is the most appropriate technique.

All the patients ultimately underwent surgery, even though we have experienced a significant realization.

## Case presentation

**Figure 1:**
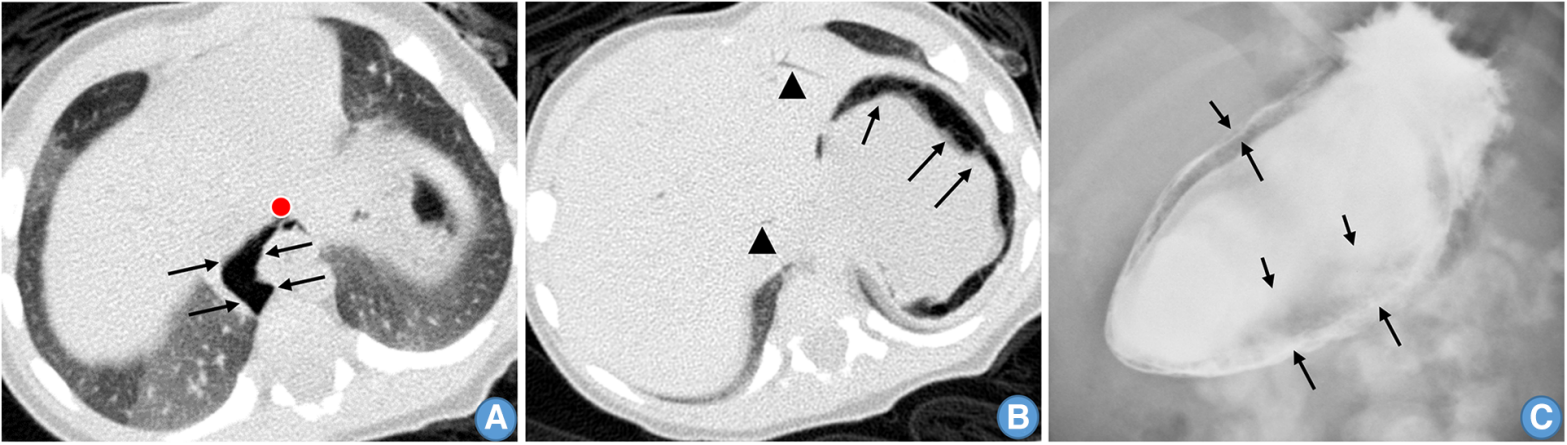
(**A**) Gas around the esophagus. (**B**) Gas is present in the stomach wall (arrows) and portal vein (triangles). (**C**) Imaging (x-ray) shows a large amount of gas in the stomach wall (arrows).

A 56-day-old male infant was admitted to the hospital because of severe vomiting without bile for 20 days and growth retardation for 10 days. The blood routine of the patient showed slightly higher white blood cells than normal. Hyponatremia, and the other blood tests showed no special abnormalities. In this report, we present a baby who had been diagnosed with pneumonia (aspiration pneumonia caused by vomiting secondary to CHPS) in a local hospital with chest CT indications of gas accumulation in the esophagus, gastric wall and portal vein (Figure 1). Therefore, NEC was considered first, and conservative treatment was started at the same time. The treatment was initially focused on treating the pneumonia and NEC, but the patient's vomiting persisted. A CHPS diagnosis was obtained with abdominal ultrasonography and tract contrast examination at this time.

**Figure 2:**
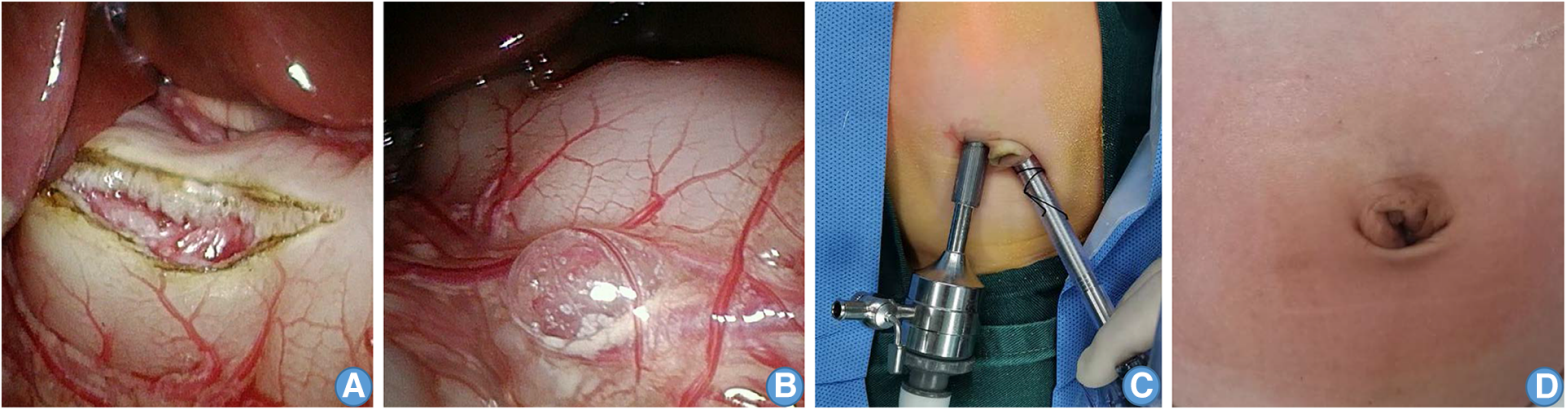
Preoperative and postoperative photographs. (**A**) laparoscope single-site surgery in the umbilical region. (**B**) Omental sac pneumatosis. (**C**) Laparoscopic pyloromyotomy during the operation. (**D**) The cut healed without leaving evident scars.

A 29-day-old male infant presented with projectile vomiting without bile. Electrolyte tests showed hyponatremia, but other tests did not have a characteristic result; sometimes the vomit resembled coffee grounds; and the infant had growth retardation. Imaging revealed gas accumulation around the wall of the gastric wall and portal vein, and a diagnosis of CHPS was made. This is a rare imaging manifestation of the disease. Repeated examination by the doctor showed no signs of peritonitis. The patient was fasted for 2 days and then underwent laparoscopic pylorotomy, after which the child's symptoms disappeared, and his growth recovered and quickly reached the level of children of the same age (Figure 2).

## Discussion

CHPS is characterized by typical symptoms of vomiting that is bile free (1). Vomiting is one of the most common symptoms for infants and children (2). However, not all patients have atypical systems and signs. The patient of case 1 was given fasting treatment for 1 week in a primary hospital because of concerns about the possibility of NEC. In case 2, due to the lack of understanding of gastric wall gas, the operation for CHPS was performed after 2 days of observation without signs of peritonitis, which also caused a certain delay in initiating the operation. The examination methods mainly rely on ultrasound and gastrointestinal contrast. At present, with the popularization of ultrasound examination technology and equipment, most of the ultrasound departments in primary hospitals are familiar with the diagnosis of CHPS (3). A pyloric length >16 mm, a pyloric muscle thickness ≥4 mm, and a pyloric tube diameter ≥14 mm are used as the diagnostic criteria. If the above three criteria were not met at the same time, a narrowness index greater than 50% could be used as one of the diagnostic criteria. Imaging examination combined with typical clinical manifestations can obtain an accurate diagnosis in most cases.

Portal vein gas is a marker of NEC in infants, especially in preterm infants (4, 5). Therefore, in the two cases, the gas in the gastric wall and portal system still needed to be differentiated from NEC. Most NEC patients have a history of abdominal distension, and the lesions are mainly located in the ileum or colon, often accompanied by positive abdominal signs. The cases in our report were accompanied by gas in the stomach wall and portal vein, but there were different abdominal signs. There is no gastric wall gas in NEC, and portal vein gas could signal a severe NEC case. Therefore, NEC with portal venous gas tends to have abdominal discomfort. A careful physical examination is necessary.

It is also necessary to understand the causes of the formation of gas (6). Because of the high tension of the stomach, the gas in the gastric mucosa enters the muscular layer of the gastric wall and subserosa. The gas may enter the omentum, esophageal serosa, and portal vein (7). However, the thickness of the muscular layer of the gastric wall and esophagus is greater than that of the small intestine and colon, which causes no full-thickness perforation and only subserosal gas, so there is no peritonitis. Therefore, there are differences in clinical manifestations and imaging examinations. The intraoperative finding of gastrointestinal gas in CHPS is not a contraindication to surgery, and the operation can be performed normally, without waiting and delaying surgery. At present, laparoscopic pyloromyotomy is the gold standard for treatment. At present, the average operation time of our center is approximately 17 min at two holes in a single site.

## Conclusion

Pneumatosis of the gastrointestinal tract, especially gas in the stomach wall, may also be a reference for the diagnosis of CHPS. This is not a contraindication to surgery, and surgery can be performed normally.

## Data Availability

The original contributions presented in the study are included in the article/Supplementary Material, further inquiries can be directed to the corresponding author.
